# Evaluation of the influence of the antibiotic ciprofloxacin in the development of an Old World screwworm fly, *Chrysomya putoria*

**DOI:** 10.1093/jis/14.1.3

**Published:** 2014-01-01

**Authors:** Adriana C. P. Ferraz, Daniele L. Dallavecchia, Débora Cardoso da Silva, Rafaela Pereira de Carvalho, Renato Geraldo da Silva Filho, Valéria M. Aguiar-Coelho

**Affiliations:** 1 Universidade Federal Rural do Rio de Janeiro, Curso de pós-graduação. Rod. BR 465, Km 7, CEP 23890-000 Seropédica, RJ, Brazil; 2 Universidade Federal do Estado do Rio de Janeiro, Rua Frei Caneca, 94, Centro, CEP 20211-040 Rio de Janeiro, RJ, Brasil; 3 Universidade Estadual do Sudoeste da Bahia, Departamento de Estudos Básicos e Instrumentais, Praça Primavera s/n, Bairro Primavera, CEP 45700-000, Itapetinga, BA, Brasil

**Keywords:** blowflies, drug, maggots, pupae

## Abstract

*Chrysomya putoria* (Wiedemann) (Diptera: Calliphoridae), an Old World screwworm fly, is a species with potential for maggot therapy practice and has been described in myiasis and forensic entomology studies. The objective of the present study was to assess the action of different ciprofloxacin concentrations on the growth and development of *C. putoria* . First instar maggots of the third generation were raised on 60 g of chicken gizzard homogenate in 65% agar diet and received ciprofloxacin chloridrate. Each concentration of the antibiotic tested (3.33 µg/mL, 6.66 µg/mL, and 13.33 µg/mL) and the control (no antibiotic) were replicated four times (40 maggots/replication). The control received distilled water instead of the antibiotic. Maggots were kept in an acclimatized chamber at 30° C during the day and 28° C at night, with 70 + 10% RH and a 14:10 L:D photoperiod. They were weighed in batches of five and stored in test tubes sealed with nylon fabric and elastic. Microsoft Excel and STAT were used for the analysis. The variation among the maggot weight means and the duration of the maggot stage, pupal stage, and time to total development (neolarvae to adult) were analyzed by Student’s *t* -test (α= 5%). The viabilities and the normality rates were compared using ANOVA, and the expected sex ratio frequency was tested by the chisquared test (χ ^2^ ). There was no significant difference among the four treatments regarding mean individual maggot weight, mean duration of the maggot inoculation until abandonment, the duration of the maggot and pupal stages, and the total duration of all stages. The sex ratios found in the four treatments did not differ from the expected. Only treatment 2 (6.66 µg/mL concentration of ciprofloxacin) differed significantly from the control in maggot and total viability. The antibiotic did not seem to alter *C. putoria* development in the postembryonic period.

## Introduction

Antimicrobial agents are medications used to eliminate different genera of microorganisms. Among the different genres of existing antimicrobials, some of the most commonly used are norfloxacin, ciprofloxacin, nitrofu-rantoin, ampicin, and sulfametoxazol/trimetropin. Ciprofloxacin is a wide-spectrum therapeutic chemical used to treat pathologies caused by gram-positive and gram-negative sensitive microorganisms ( DEF 2001 ; Korolkovas 2002 ; Martindale 2002 ) and is appropriate for the treatment of infections caused by pathogens sensitive to the drug, such as infections of the upper respiratory tract, ear, nose, throat, maxillofacial area, urinary and kidney tracts, gastrointesti-nal tract (including typhoid fever), bile duct, soft tissues and infected wounds, bones and joints, gynecological and obstetric infections, septicemia, meningitis, and peritonitis, and can be used to treat imminent risk of infection (prophylaxis) in patients with immune suppression (Quinoflox 2011)

Many wounds, such as those mentioned above, can be treated alternatively with the use of maggot therapy, also known as maggot debridement therapy, biotherapy, or biosurgery, which consists of applying live fly maggots (Diptera: Calliphoridae) to wounds that do not heal with the objective of removing the necrotic material and promot-ing new tissue growth. Its use and populari-zation have increased in many countries due to the great efficacy of the maggot in removing necrotic material and its safe and easy application ( Sherman et al. 2000 ; Martini and Sherman 2003 ; Marcondes 2006 ; Dal-lavechia et al. 2010, 2011). *Chrysomya putoria* (Wiedemann) (Diptera: Calliphoridae) is easily obtained throughout the Brazilian territory, it is easy and cheap to rear, its maggots develop quickly, and its behavior and biology are compatible with use in maggot therapy ( Marcondes 2006 ; Oliveira-Costa 2011). In maggot therapy, it is important to know how antibiotics used in conjunction with maggots act on the maggots because the antibiotics can influence the natural activities and survival of the maggots.

Patients undergoing myiasis often use antibiotic therapy in association with myiasis because they might have other associated infirmities and skin wounds that are very contaminated ( Ferraz et al. 2008 . 2010), therefore the use of antibiotics would help the healing process because antibiotics help in disinfection. So, it is necessary to know the effects of these antibiotics on the maggots.

Necrophage insect development rates can be affected by substances introduced to the insect through its food ( Introna et al. 2001 ), and this can affect studies on these insects, such as those linked to the post mortem interval estimate in forensic entomology, because this analysis is based on the development period of these insects ( Estrada et al. 2009 ).

*Chrysomya putoria* is a species with potential for maggot therapy practice and has also been described in myiasis and forensic entomology studies. The objective of the present study was to assess the action of different antibiotic concentrations on the growth and development of the species.

## Materials and Methods

The dipterans used throughout the experiment were reared in the Dipterans Study Laboratory, Department of Microbiology and Parasitology, Federal University of the State of Rio de Janeiro.

The *C. putoria* colony was started with adults collected in a zoological garden located in the Quinta da Boa Vista, São Cristóvão, Rio de Janeiro. Three traps were used, following the model of Mello et al. (2007) , and they contained sardine as bait. The traps were exposed for approximately five hours in the morning. After collecting dipteran adults and maggots *,* the insects were taken to the laboratory, where they were screened and identified taxonomically according to Mello et al. (2003) . The flies were reared in transparent plastic cages (40 x 30 x 20 cm) with an opening in the upper part for airing and a front opening for access to the inside of the cage, which was covered with nylon fabric. They were fed water, honey, and water solution (50%) and chicken gizzard or beef as a protein source, oviposition substrate, and aid in ovary maturation.

The 1 ^st^ instar maggots of the third laboratory generation were transferred using a fine brush to 100 mL glass beakers each containing 60 g of chicken gizzard homogenate in a 65% agar diet ( Ferraz et al. 2012 ). The diet was selected because it was practical to homogenize substances to be tested and it was sterile (autoclaved), which makes the antibiotic completely available to act only on the maggot. The antibiotic used was 400 mg Hifloxan (ciprofloxacin chloridrate, Halexistar, www.halexistar.com.br ). Each replication received 1 mL of the antibiotic at three different concentrations, resulting in the following concentrations in the diet: 3.33 µg/mL, 6.66 µg/mL, and 13.33 µg/mL. The concentrations were chosen from ciprofloxacin serial concentration (500 mg via oral administration 12 times in 12 hr: 2.97 µg /mL, 400 mg via intravenous administration 12 times in 12 hr: 4.56 µg /mL). The first concentration chosen (T1) would have an approximate value between the oral and intravenous serum concentrations, the second concentration (T2) would be approximately double the first, and the third concentration (T3) would be approximately four times as great as T1. Each concentration of the antibiotic and the control were replicated four times with 40 maggots in each replication. The control received distilled water in place of the antibiotic.

Each beaker of each replication was inserted in a larger beaker (400 mL) containing steri-lized sawdust to allow maggot pupation when the maggot was mature after abandoning the diet, and the beaker was sealed with nylon fabric and elastic. The maggots were kept in an acclimatized chamber at 30° C during the day, 28° C at night, 70 ± 10% RH, and with a 14:10 L:D photoperiod. Observations were made daily at 12:00.

The maggots, after abandoning the diet, were weighed in batches of five on an analytical scale and stored in test tubes sealed with nylon fabric and elastic to observe adult emergence. The dates of pupation and adult emergence were recorded along with the sex ratio and adult morphological abnormalities.

Microsoft Excel ( www.microsoft.com ) was used for the gross data analysis and the PAST program ( http://folk.uio.no/ohammer/past/ ) for the other analyses. The variation among the maggot weight means and the durations of the maggot stage, pupal stage, and the time to total development (neolarvae and adults) were analyzed using Student’s *t* -test(α= 5%). The viability and normality rates were compared by ANOVA. The sex ratio was tested in relation to the expected frequency by the chisquare test (χ ^2^ ).

## Results

There was not a significant difference among the four treatments in mean individual maggot weight ( Table 1 ), and there was not a significant difference among the four treatments in both the mean duration of the inoculation of the maggots until abandonment and the duration of the maggot stage, pupal stage, and the time to total development (days) ( Table 2 ).

**Table 1.**
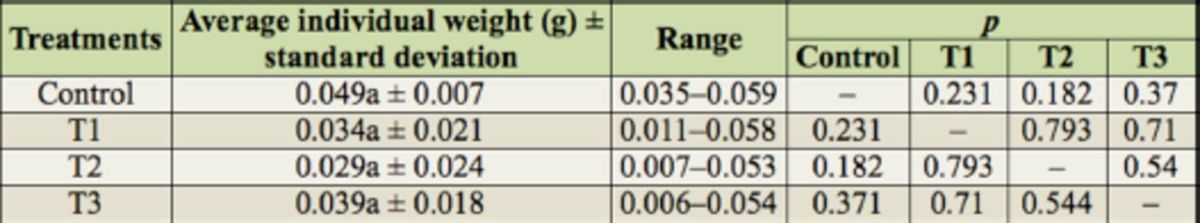
Maggot weight (g) of *Chrysomya putoria* from four treatments with different ciprofloxacin concentrations reared in an acclimatized chamber (30º C day, 28° C night, 70 + 10% RH. 14:10 L:D photoperiod).

Means followed by the same letter do not differ significantly by the *t* -test (α= 5%). Control = chicken gizzard homogenate in 65% agar diet; T1 = chicken gizzard homogenate in 65% agar diet + 3.33 μg/mL ciprofloxacin; T2 = chicken gizzard homogenate in 65% agar diet + 6.66 μg/mL ciprofloxacin, T3 = chicken gizzard homogenate in 65% agar diet + 13.33 μg/mL ciprofloxacin.

**Table 2.**
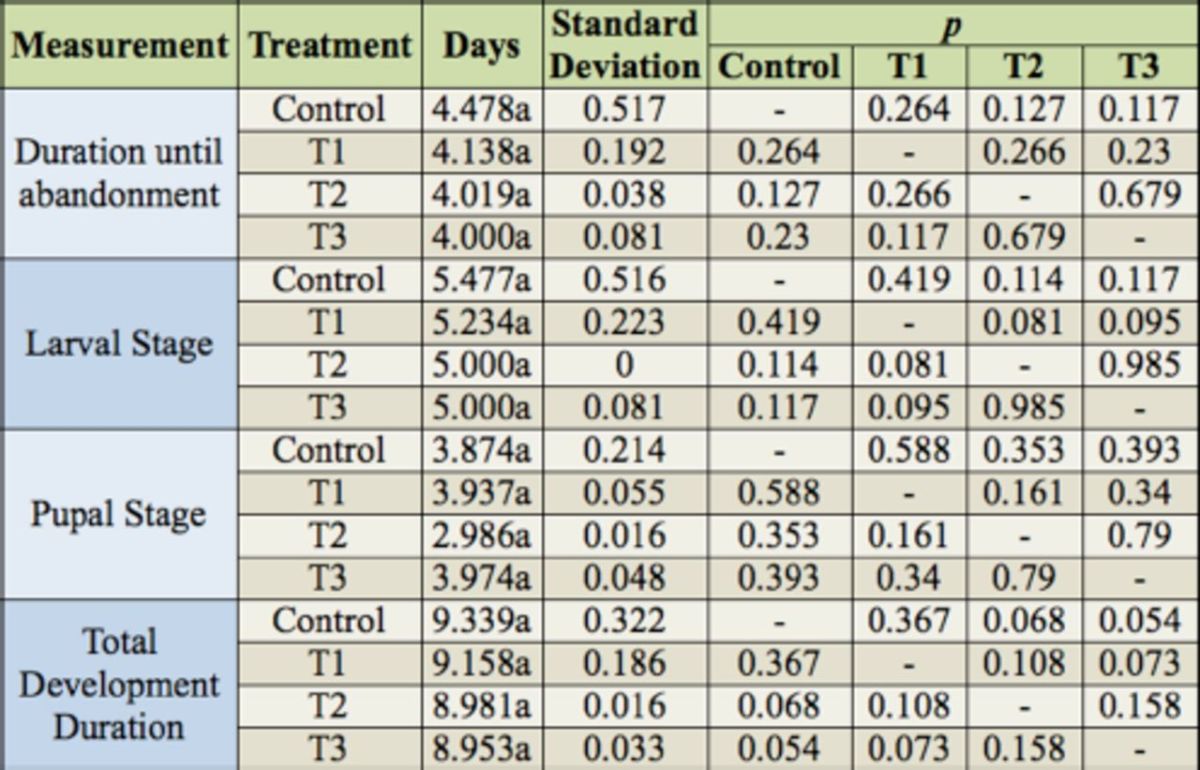
Mean duration of the postembryonic development stages (days) of *Chrysomya putoria* from four treatments with different ciprofloxacin concentration reared in an acclimatized chamber (30º C day, 28° C night, 70 + 10% RH, 14:10 L:D photoperiod).

Means followed by the same letter do not differ significantly by the *t* test (α=5%). Control = chicken gizzard homogenate in 65% agar diet; T1 = chicken gizzard homogenate in 65% agar diet + 3.33 μg/mL ciprofloxacin; T2 = chicken gizzard homogenate in 65% agar diet + 6.66 μg/mL ciprofloxacin, T3 = chicken gizzard homogenate in 65% agar diet + 13.33 μg/mL ciprofloxacin.

**Figure 1.**
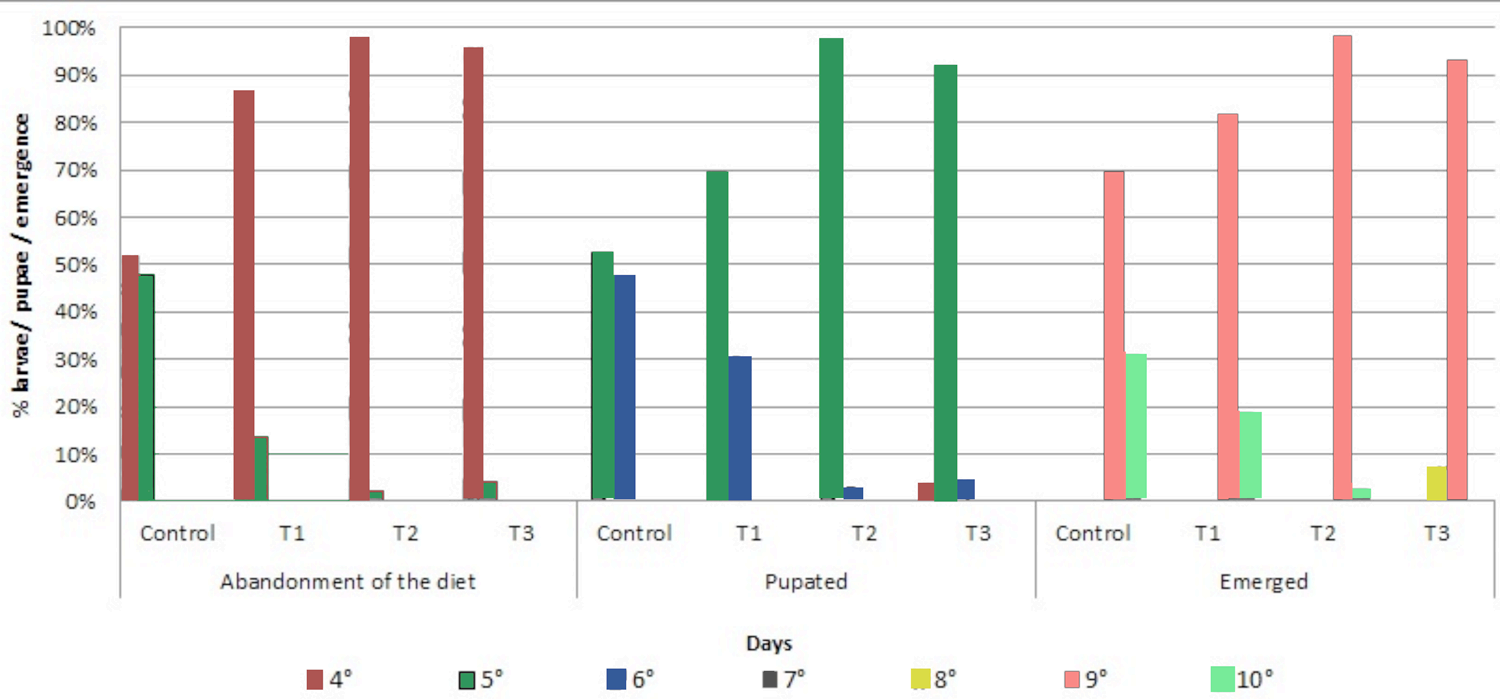
Diet abandonment rhythm of *Chrysomya putoria* maggots at pupation and emergence (%) from four treatments (40 maggots/replication): control = chicken gizzard homogenate in 65% agar diet; T1 = chicken gizzard homogenate in 65% agar diet + 3.33 μg/mL ciprofloxacin; T2 = chicken gizzard homogenate in 65% agar diet + 6.66 μg/mL ciprofloxacin; T3 = chicken gizzard homogenate in 65% agar diet + 13.33 μg/mL ciprofloxacin *.* High quality figures are available online.

Figure 1 shows the diet abandonment rhythm of the *C. putoria* maggots and the pupation and emergence percentages in the four different treatments (control: chicken gizzard homogenate in 65% agar diet; T1: chicken gizzard homogenate in 65% agar diet plus 3.33% ciprofloxacin; T2: chicken gizzard homogenate in 65% agar diet plus 6.6% ciprofloxacin, T3: chicken gizzard homogenate in 65% agar diet plus 13.33% ciprofloxacin). Diet abandonment peaked in the control on the fourth and fifth days, while in the treatments with the antibiotic it peaked predominantly on the fourth day. The control pupation peaked on the fifth and sixth days, while the other treatments peaked on the fifth day. Emergence peaked on the ninth day in all the treatments.

**Figure 2.**
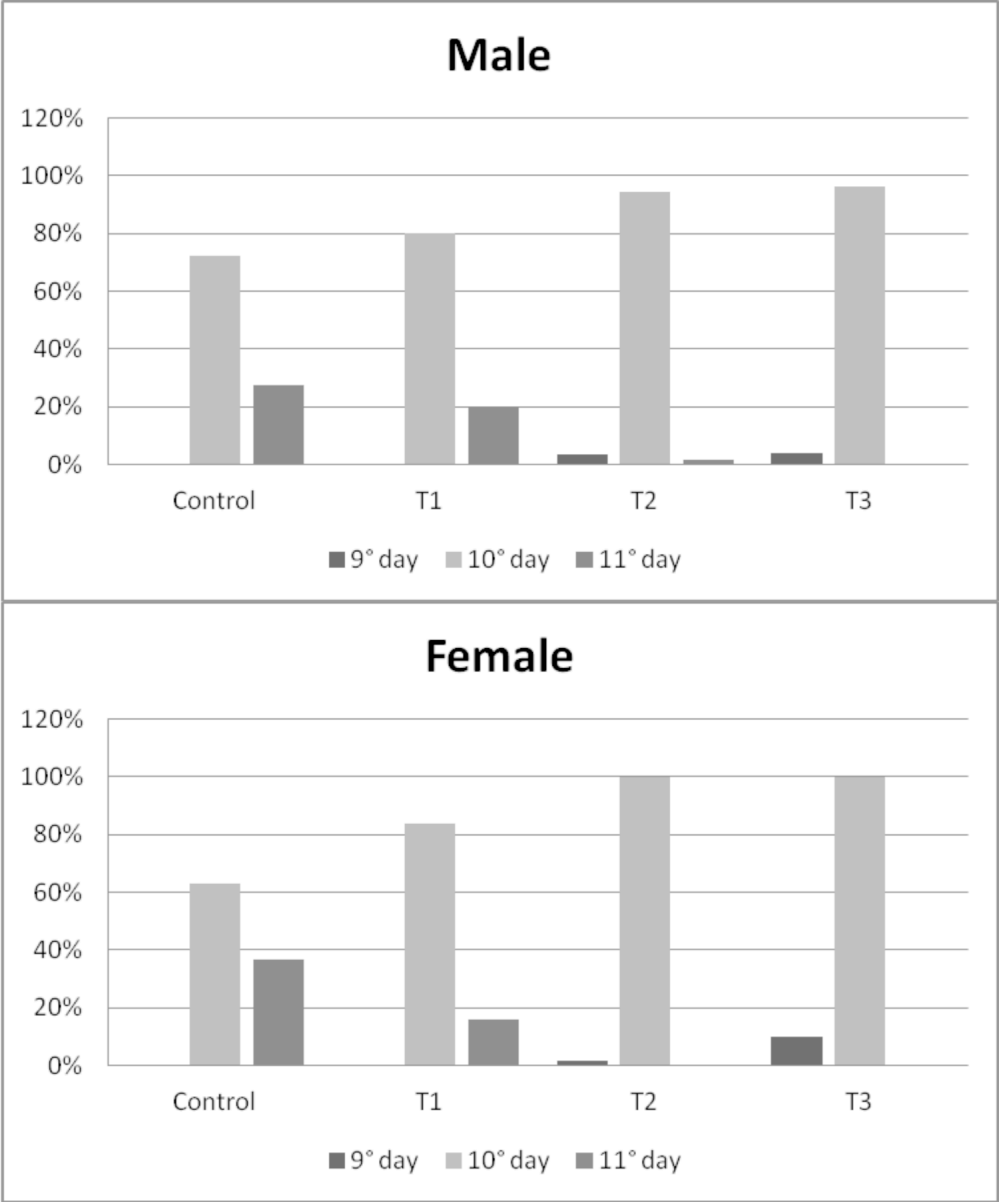
Emergence rates (in days) of male and female *Chrysomya putoria* reared on four treatments: control = chicken gizzard homogenate in 65% agar diet; T1 = chicken gizzard homogenate in 65% agar diet + 3.33 μg/mL ciprofloxacin; T2 = chicken gizzard homogenate in 65% agar diet + 6.66 μg/mL ciprofloxacin; T3 = chicken gizzard homogenate in 65% agar diet + 13.33 μg/mL ciprofloxacin. High quality figures are available online.

The *C. putoria* male and female emergence rates by days were similar ( Figure 2 ). The sex ratios found were: control: males = 47%, females = 53%; T1: males = 55%, females = 45%; T2: males = 50%, females = 50%; T3: males = 44%, females = 56%. The chisquare tests showed that the sex ratios observed in the four treatments did not differ from the expected (control: χ ^2^ = 0.398; T1: χ ^2^ = 0.909; T2: χ ^2^ = 0.009; T3: χ ^2^ = 1.923, χ ^2^ from table with 1 df α=5% (5% probability) is 3.84.

Only the control produced100% normal adults (T1 = 96%, T2 = 98%, T3 = 99%). However, there was no significant statistical difference among the treatments (control x T1: *p* = 0.192; control x T2: *p* = 0.073; control x T3; *p* = 0.356; T1 x T2: *p* = 0.408; T1 x T3: *p* = 0.249; T2 x T3: *p* = 0.463). Regarding the maggot, pupa and total viabilities, only treatment 2 differed significantly from the control in the maggot and total viabilities ( Table 3 ).

**Table 3.**
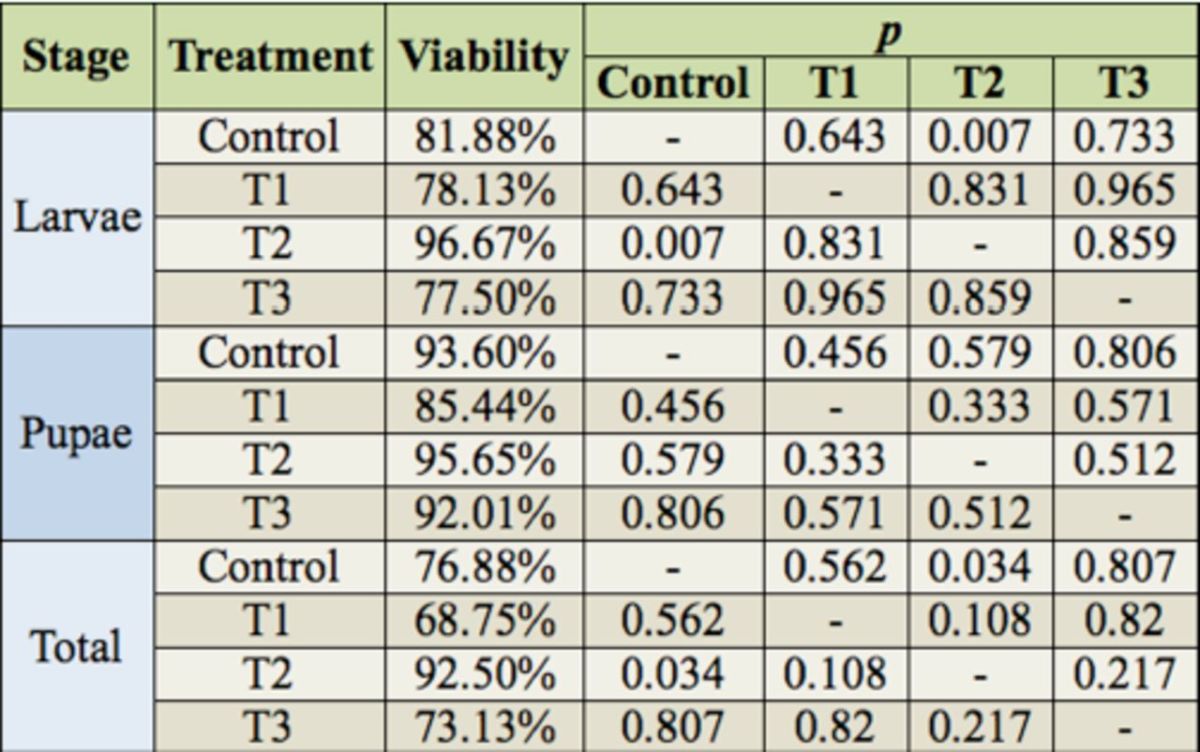
Comparison by ANOVA of the viability of the maggots, pupae, and total development (neomaggot to adult) of *Chrysomya putoria* from four treatments with different ciprofloxacin concentrations reared in an acclimatized chamber (30º C day, 28° C night, 70 + 10% RH, 14:10 L:D photoperiod).

Control = chicken gizzard homogenate in 65% agar diet; T1 = chicken gizzard homogenate in 65% agar diet + 3.33 μg/mL ciprofloxacin; T2 = chicken gizzard homogenate in 65% agar diet + 6.66 μg/mL ciprofloxacin, T3 = chicken gizzard homogenate in 65% agar diet + 13.33 μg/mL ciprofloxacin.

## Discussion

Different concentrations of the same antibiotic need to be tested because the same antimicrobial added to a diet may be safe, inhibitory, or highly damaging depending on the concentration used ( Singh and House 1970 ). In our study, more than 1 g of diet was provided for each maggot because this amount was reported by Aguiar-Coelho and Milward-de-Azevedo (1996) as being ideal when using meat in different caliphoridae. Care was taken so that the density of food per larvae did not interfere in the assessment of the efficiency of these diets by causing stress due to competition for resources or chemical alterations in the food substrate resulting from the maggot metabolism ( Khazaeli et al. 1993 ; Bubli et al. 1998 ). Considering that 100% normal dipterans were obtained in the control and the other values in the other treatments did not differ significantly from the control, the amount of diet per larvae can be considered adequate for laral development. Furthermore, it can be assumed that the ciprofloxacin antibiotic does not affect the phenotype. Larval density in reference to available food can influence the phenotype determination of bionomic traits and alter determining components of the adaptive value of the organisms, as reported by Ribeiro et al. (1993) , Reis et al. (1994) , Roper et al. (1996) , and Ribeiro (1998) in studies using dipterans. When there are no variations in the symmetry, it means that sufficiently intense disturbances that could not be buffered or neutralized with metabolic adjustments to guarantee homeostatic stability in the development patterns were not produced (Lomonaco and Germanos 2001).

Although not significant, the treatments with the antibiotic presented variation intervals in individual weight with minimum values (control > T1 > T2 > T3) and maximum values (control > T1 > T3 > T2) smaller than the control. These values were also lower than those reported by Ferraz et al. (2012) , who used meat, chicken gizzard, and chicken gizzard homogenate in 65% agar diet to rear *C. putoria* . The total duration of the maggot development stage was longer in the present experiment, which is understandable, because meat is the natural diet of this species ( Leal et al. 1982 ).

Ahmad et al. (2006) compared the development of *Cochliomyia macellaria* (F.) in sterile medium and other medium with bacteria, and a faster development rate and greater survival was observed in the sterile medium than in the medium with bacteria ( Ahmad et al. 2006 ). In our study, the rhythm of the maggots abandoning the diet, pupation, and emergence tended to be earlier in the treatments with antibiotic than in the control. As the maggots used in this experiment were not sterile, they probably took bacteria to the culture media, causing the bacteria to be been neutralized in the culture media with antibiotics but not in the control.

The main period of food resource limitation in Calliphoridae is in the maggot period, when they ingest the maximum amount of food in the shortest time interval (Goodbrod and Goff, 1990). A longer feeding period can occur to compensate the poor nutritional quality of the food (Kamal 1958; Levot et al. 1979; Paes et al. 2000). In the present study, the shorter the time the insects stayed on the diet (until abandonment), the lower their weight (control > T1 > T3 > T2)

The gender rates of the individuals in the four different treatments in the present study indicated stability in the population according to the chisquare analysis and the concepts by Fisher (1930) : only when the sex ratio is 1:1 will there be stability in the population; a sex ratio with deviation is not evolutionarily stable, because in future generations there will be gradual increases in the proportion of the sex observed in smaller number.

The ciprofloxacin antibiotic did not alter the mean maggot weight of *C. putoria* , the duration of the postembryonic stages, or the sex ratio. Some studies on the effects of other chemical substances on *C. putoria* maggot development have reported different results. Carvalho (2004) studied the effect of cocaine, exposing liver containing the substance, and observed that *C. putoria* maggots exposed to the drug developed significantly faster compared to the control. It was also reported that the maggot exposed to liver containing cocaine started and ended the pupation process before the control, but emergence time was similar.

Another substance that accelerated *C. putoria* maggot development was diazepam, and the maggots treated with diazepam were heavier than the control maggots ( Carvalho et al. 2001 ). The amphetamine anphepramon also accelerated *C. putoria* development by approximately 48 hours ( Carvalho, 2004 ).

The analgesic scopolamine retarded development in groups with higher concentration ( Grella et al. 2007 ), and the amphetamine increased the *C. putoria* pupation period ( Nitsche 2010 )

In the present study, ciprofloxacin only significantly altered the maggot and total viabilities in T2. The T2 viabilities were highest, but those of the other treatments were also high. In dipterans, viability of over 60% is considered promising ( Loureiro et al. 2005 ). There is also the possibility that the maggots did not metabolize the drug, as reported by Grella and Thyssen (2008) in a study on immature *C. albiceps* , *C. megacephala* , and *C. putoria* reared on artificial diet with the addition of oxycodone chloridrate where significant differences were not observed in the development among the control and experimental groups.

Insects absorb substances used by the humans the insects are feeding on, and toxins and controlled substances have been detected both in the insect and the quitinized remains removed from victims at an advanced stage of decomposition ( Goff and Lord 2001 ). Thus, it is necessary to study the effects that drugs and toxins exercise on maggot development because acceleration or delay in development can interfere in the postmortem interval estimates based on the biology of these insects ( Goff et al. 1992 ; Carvalho et al. 2001 ; Grella and Thyssen 2008 ). In Campinas, São Paulo, Brazil, from 1993 to 1998, *C. putoria* was among the most frequent insects in morgue and field collections and was also considered to be a forensic indicator for the area ( Carvalho et al. 2000 ).

Sherman et al. (1995) carried out an experiment on the action of seven antibiotics on *Lucilia sericata* maggot and pupae growth and development, as *L. serrata* is used in maggot therapy. The drugs they used belonged to the penicillin, cephalosporin, and aminoglycoside classes. These antibiotics were added to agar-liver culture medium at several concentrations, and one-day-old maggots were placed in the medium. The authors concluded that the antibiotics did not interfere with maggot growth or maturation, a very similar finding to findings of our study. *Chrysomya putoria* maggots have been used successfully for maggot therapy in Wistar rats, but these maggots have not yet been used in humans in Brazil because more studies need to be done ( Nitsche 2010 ). Therefore, studies such as the present will help in using *C. putoria* in forensic entomology, maggot therapy, and clinical myiasis.
